# Isolation, crystal structure determination and cholinesterase inhibitory potential of isotalatizidine hydrate from *Delphinium denudatum*

**DOI:** 10.1080/13880209.2016.1240207

**Published:** 2016-12-29

**Authors:** Hanif Ahmad, Shujaat Ahmad, Ezzat Khan, Adnan Shahzad, Mumtaz Ali, Muhammad Nawaz Tahir, Farzana Shaheen, Manzoor Ahmad

**Affiliations:** a Department of Chemistry, University of Malakand, ChakdaraKP, Pakistan;; b Department of Pharmacy, Shaheed Benazir Bhutto University, Sheringal, KP, Pakistan;; c Department of Physics, University of Sargodha, Sargodha, Punjab, Pakistan;; d HEJ Research Institute of Chemistry, International Center for Chemical and Biological Sciences (ICCBS) University of Karachi, Karachi, Pakistan

**Keywords:** Diterpenoid alkaloid, X-ray structure, DFT calculations, acetylcholinesterase (AChE) and butyrylcholinesterase (BChE) inhibition

## Abstract

**Context:***Delphinium denudatum* Wall (Ranunculaceae) is a rich source of diterpenoid alkaloids and is widely used for the treatment of various neurological disorders such as epilepsy, sciatica and Alzheimer’s disease.

**Objective:** The present study describes crystal structure determination and cholinesterase inhibitory potential of isotalatazidine hydrate isolated from the aerial part of *Delphinium denudatum*.

**Materials and methods:** Phytochemical investigation of *Delphinium denudatum* resulted in the isolation of isotalatazidine hydrate in crystalline form. The molecular structure of the isolated compound was established by X-ray diffraction. The structural data (bond length and angles) of the compound were calculated by Density Functional Theory (DFT) using B3LYP/6-31 + G (p) basis set. The cholinesterase inhibitory potential of the isolated natural product was determined at various concentrations (62.5, 125, 250, 500 and 1000 μg/mL) followed by molecular docking to investigate the possible inhibitory mechanism of isotalatazidine hydrate.

**Results:** The compound crystallized in hexagonal unit cell with space group *P*6_5._ Some other electronic properties such as energies associated with HOMO-LUMO, band gaps, global hardness, global electrophilicity, electron affinity and ionization potential were also calculated by means of B3LYP/6-31 + G (p) basis set. The compound showed competitive type inhibition of both acetylcholinesterase (AChE) and butyrylcholinesterase (BChE) with IC_50_ values of 12.13 μM and 21.41 μM, respectively.

**Discussion and conclusion:** These results suggest that isotalatazidine hydrate is a potent dual cholinesterase inhibitor and can be used as a target drug in Alzheimer diseases. This is first report indicating isotalatazidine hydrate with anticholinesterase potential.

## Introduction

*Delphinium denudatum* Wall. (Ranunculaceae) also known as Jadwar is an annual herb and its length varies from 40 to 80 cm. It is found at high altitude habitats ranging from 2400 to 3650 m in the western Himalayas (Murray 1984). The roots of this plant are traditionally used for the treatment of fungal infections, epilepsy, paralysis, cholera, jaundice and cardiac diseases (Atta-ur-Rahman et al. 1997; Choudhary 1999; Raza et al. 2003). The genus *Delphinium* is a rich source of biologically efficient compounds bearing more complicated structures which are generally diterpenoid and norditerpenoid alkaloids (Benn & Jacyno et al. 1983). Some species of the genus have shown insecticidal or antirheumatics activities and are also reported for the treatment of sciatica (Baytop 1999). The crude extract obtained as aqueous and acetone fractions is biologically active and possess anti-epileptic activity (Raza et al. 2001). Recently a number of alkaloids have been reported from *Delphinium* and *Aconitum* (Ranunculaceae) species (Atta-ur-Rahman et al. 2000; Shaheen et al. 2005, 2006, 2015). To determine the exact fraction of the crude extract against a certain disease is a key challenge in natural product research. In order to get the expected results, isolation followed by identification is the main step. Based on the mechanism and the factor responsible for certain diseases natural products are tested and their activities are recorded.

Alzheimer’s disease (AD) is one of the most common types of dementia with a neurodegenerative condition of multifactorial nature. The cholinergic brain synapses and neuromuscular junctions contains AChE (EC 3 1.1.7). This enzyme is mainly important for hydrolysis of the acetylcholine (Perry et al. 1987). It is believed that memory impairments in senile dementia diseases are because of an abnormal decrease in brain activity of cholinergic function (Yu et al. 1999). The patho-physiology of AD suggests that the loss of cognition ability is related to the continuous decrease in the level of acetylcholine resulting from the degeneration of cholinergic neurons. The situation is further deteriorated by the decomposition of already available acetylcholine by AChE in synaptic region of neuronal cells. Thus, the inhibition of AChE can compensate the deficiency of acetylcholine and can improve cognitive abilities. In extreme cases of AD, the AChE level may decline up to 90% as compared to the healthy brain (Arendt et al. 1992; Greig et al. 2001) making it uncontrollable in later stages of AD. However, several studies (Darvesh et al. 2003; Grossberg 2003) have shown the enhanced levels of iso-enzyme butyrylcholinesterase (BChE) in the brains of AD patients which is appropriate for cognition. Though, strong evidence has shown that in advanced AD, the BChE compensates for the loss of neuronal AChE and hence performs the function of AChE. Another study elsewhere reveals that mice in AChE knockout mouse model (Mesulam et al. 2002) did not suffer from cholinergic hyper activation in the absence of AChE as acetylcholine hydrolysis was controlled by BChE (Sawatzky et al. 2016). Several reports reveal that considerable amount of BChE is accumulated in Alzheimer’s plaques as compared with plaques present in normal brains. The exploration of natural cholinesterase inhibitors is a challenging task in the field of drug development, especially for the treatment of Alzheimer’s and other related diseases (Atta-ur-Rahman et al. 2002).

To the best of our knowledge, the solid state structure determination, comparative DFT study, cholinesterase inhibition activity and molecular docking studies of isotalatizidine hydrate crystal is reported here in for the first time.

## Materials and methods

### Chromatography

Pre-coated aluminum sheets were purchased and used for TLC (G-60, F-254). TLC plates were visualized in UV light (254 and 366 nm) and by Dragendorff's reagent.

### Plant material

The aerial parts of *D. denudatum* were collected from its natural high altitude habitat in Swat, KP, Pakistan, in the month of May 2012. The plant was identified by Prof. Dr. M. Nisar, Plant taxonomist, Botany department, University of Malakand and a voucher specimen no. H.UOM.BG-160 was deposited at the herbarium of Botany Department, University of Malakand. The plant material was shade dried, ground, then properly stored for further processing.

### Extraction and isolation

The dried powder of *D. denudatum* aerial parts (5 kg) was subjected to maceration with 80% methanol (3 × 10 L) at room temperature (23 ± 1 °C). Solvent was evaporated by using rotary evaporator to get 300 g crude extract. The crude methanol extract was subjected to partition between CHCl_3_ and H_2_O at acidic (pH =1–2), basic (pH =8–10) and neutral pH to get CHCl_3_ soluble fractions at different pH. The crude basic (pH =8–10) fraction (8 g) was further fractionated over column using silica gel (160 g) with elution started from *n*-hexane (non-polar system), *n*-hexane-chloroform gradients up to 20% methanol-chloroform gradient that afforded ten sub-fractions. Sub-fraction D-4 (90% CHCl_3_-hexane), on repeated FCC using *n-*hexane-acetone solvent system (95:5) containing 10 drops of DEA per 100 mL afforded isotalatizidine hydrate (15 mg) as colourless crystals.

### X-ray crystallography

X-ray diffraction crystal for a single structure was performed by using a STOE-IPDS II fitted with low-temperature unit of a Bruker kappa APEXII CCD diffractometer using Mo-Kα radiation (*λ* = 0.71073 Å) and graphite-monochromator at room temperature. Crystal structure determination and refinements were accomplished by SIR97 (Altomare et al. 1999), SHELXL97 (Sheldrick 2007) and WinGX (Farrugia 1999).

### DFT calculations

Crystallographic data were used to get optimized ground state geometry of compounds **1** using DFT method following B3LYP-631G (p) model of theory (Zhang & Musgrave 2007; Wang et al. 2007; Jacquemin et al. 2008). Electronic properties like frontier molecular orbital (HOMO-LUMO) energies, optimized geometries gap, global hardness, ionization potential, electron affinity and global electrophilicity of the compound were calculated with same methods (Wang et al. 2007). GAUSSIAN-03 program and Gauss-view molecule visualizer were used for the calculation of the abovementioned data.

### Cholinesterase inhibition assay and determination of IC_50_

AChE (Electric-eel EC 3.1.1.7), BChE (horse-serum E.C 3.1.1.8), AChI, AChCl, 5,5′-dithiobis[2-nitrobenzoic-acid] (DTNB) and galanthamine were purchased from Sigma Aldrich. All solvents used during the course of isolation and/or purification were of analytical grade. Enzyme (AChE/BChE) inhibition activities were measured by spectrophotometric method as described in literature (Ellman et al. 1961). Reported protocol and assay conditions were followed throughout (Rocha et al. 1993). AChI and AChCl substrates were used to assay AChE and BChE, respectively. DTNB was used as reagent for the measurement of cholinesterase activity. Solution containing DTNB (0.2 mM) in 62 mM sodium phosphate buffer (pH 8.0, 880 μL), test compound solution (40 μL) and AChE or BChE solution (40 μL) were mixed, incubated for 15 min (25 °C). AChE or BChE (40 μL), were added to initiate the respective solution. The hydrolysis of AChE and BChE were noted by naked eye due to formation of yellow colour 5-thio-2-nitrobenzoate anion at a wavelength of 412 nm (15 min). Reactions were performed in triplicate in a BMS spectrophotometer (USA) and the results presented are average values. The concentrations of the compound that inhibited the hydrolysis of substrates (as mentioned above) by 50% (IC_50_) were determined as a function of increasing concentration of the compound in the assays on the inhibition values.

### Molecular docking

Molecular docking was done to ascertain the binding mode of the isolated compound for the competitive and noncompetitive inhibition with the target enzymes, acetylcholinesterase (AChE) and butyrylcholinesterase (BChE). MOE (Molecular Operating Environment) (www.chemcomp.com) software was used for the molecular docking studies. The three dimensional structure of the isolated compound was generated by using the builder tool of the MOE software. The isolated natural product was 3D protonated and then energy minimization of the compound was done by using the default parameters of the MOE (gradient: 0.05, Force Field: MMFF94X). The isolated compound was then saved in mdb file for further evaluation in molecular docking. The 3D structures of the two target enzymes (AChE and BChE) were downloaded from the protein databank (PDB) PDB id: 1ACL and 1POP, respectively. Each protein was opened in MOE, water molecules were removed from the proteins. Then 3D protonation of each enzyme was carried out and after 3D protonation energy minimization was done for the stability of the proteins by using the default parameters of the MOE (Alam et al. 2016).

## Results

### Crystal structure determination

**Figure 1.**
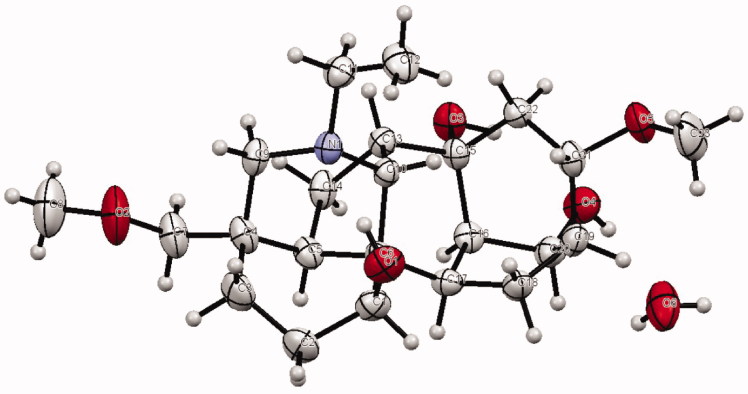
Compound **1**, thermal ellipsoid are drawn at 50% probability, showing all hydrogen atoms.

Isotalatizidine hydrate was isolated as colourless crystals from the aerial part of *D. denudatum* and its structure was determined by X-ray diffraction technique. The natural product crystallized in hexagonal crystal system with space group of *P6_5_*_._ The molecular structure has been shown in Figure 1 and the data related to crystal structure determination and refinement of the compound are enlisted in Table 1 while selected structural parameters (bond lengths and angles) have been provided in Table 2. All the bond length and angles are in the expected range (Shaheen et al. 2004).

**Table 1.** Data related to structure determination and refinement of compound **1**.

Crystal parameter	**1**
Empirical formula	C_23_H_39_NO_6_
Formula weight	425.
Temperature (K)	296
Wavelength (Å)	0.71073
Crystal system	Hexagonal
Space group	*P6_5_*
a	10.70(3)
c	34.28(1)
Volume Å^3^	3399(2)
μ (mm^−1^)	0.09
Z	4
Density (Mg m^−3^)	1.247
(h, k, l) min	(-12, -13, -35)
(h, k, l) max	(13, 10, 44)
Theta (max)	27.6
R (reflection)	0.062(4536)
wR_2_	0.170

**Table 2.** List of selected bond lengths (Å) and bond angle (^o^) of compound **1**.

Experimental	B3LYP-631G		Experimental		B3LYP-631G
List of bond lengths					
O1-C1	1.455(6)	1.436	C21-O5	1.448(6)	1.429
O2-C8	1.411(8)	1.423	C15-C16	1.563(6)	1.531
O3-C15	1.450(5)	1.436	C16-C20	1.534(6)	1.580
O4-C20	1.428(6)	1.418	C16-C17	1.552(6)	1.584
N1-C9	1.486(6)	1.470	C18-C19	1.549(6)	1.530
N1-C11	1.490(6)	1.466	C17-C18	1.562(6)	1.622
N1-C10	1.499(5)	1.526	C19-C21	1.547(7)	1.560
O5-C23	1.410(6)	1.421			
List of bond angles					
C8-O2-C7	113.6(5)	115.61	C13-C15-C22	111.9(3)	114.95
C23-O5-C21	114.4(4)	116.08	O3-C15-C16	108.1(3)	111.04
C9-N1-C11	110.4(3)	112.29	C17-C16-C15	113.5(3)	115.05
C9-N1-C10	112.3(3)	114.02	C20-C16-C15	109.8(3)	112.52
O1-C1-C2	110.8(4)	112.09	C16-C17-C18	103.6(3)	105.53

#### Optimized geometry of isotalatizidine hydrate

**Figure 2.**
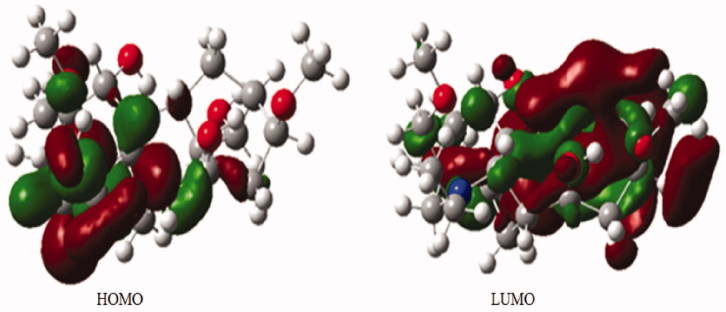
HOMO-LUMO of compound **1** calculated at B3LYP/6-31 + G (p).

To obtain additional details about the structure of isotalatizidine hydrate, DFT calculations were performed. The structure of the compound **1** with proper orientation was optimized following B3LYP-631G (p) basis set. The optimized geometry is shown in Figure 2. The calculated energy parameters of the compound are given in Table 3.

**Table 3.** Energy parameters of the compound **1**.

	**1**
Є_HOMO_ (eV)	−0.166
Є_LUMO_ (eV)	0.054
ΔЄ = (Є_LUMO-_Є_HOMO_) (eV)	0.220
IE= -Є_HUMO_ (eV)	0.166
EA= -Є_LUMO_ (eV)	−0.054
Global hardness (η) = 1/2 (Є_LOMO_-Є_HOMO_)	0.11
Chemical potential μ = 1/2 (Є_HOMO_+Є_LUMO_)	−0.056
Global electrophilicity ω = μ^2^/2η	0.014

#### Cholinesterase inhibition

**Figure 3.**
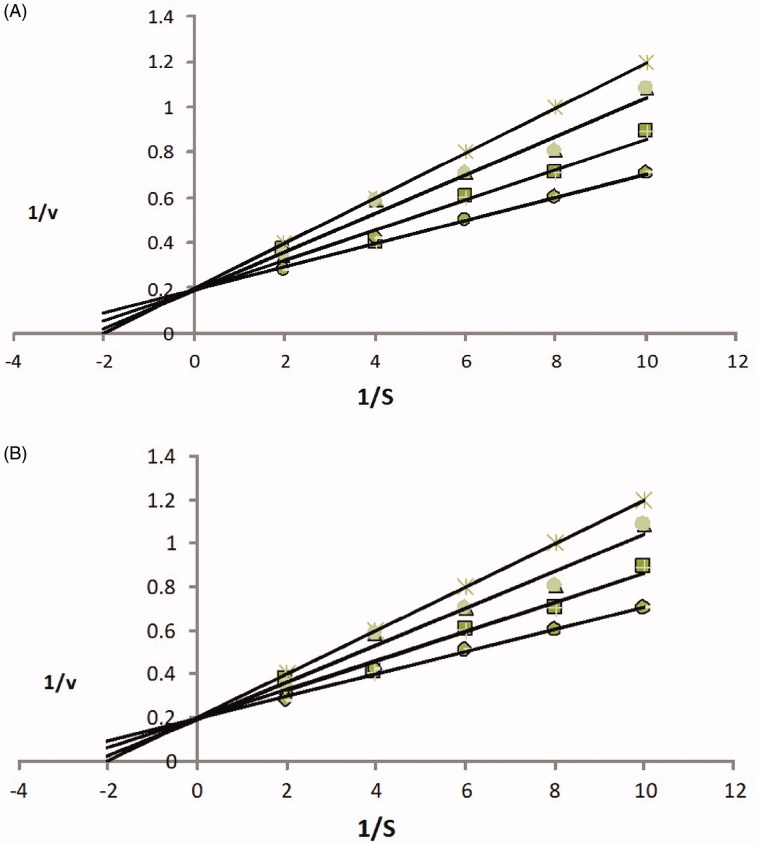
(A) Acetylecholinesterase inhibition by compound **1** is the Lineweaver–Burk plot of reciprocal of initial velocities versus reciprocal of four fixed substrate concentrations in absence (•) and presence of 100 μM (▪), 75 μM (▴), 50 μM (×) of compound **1**. (B) Butyrylecholinesterase inhibition by **1** in absence (•) and presence of 100 μM (▪), 75 μM (▴), 50 μM (×) of compound **1**.

Isotalatizidine hydrate was tested for its possible cholinesterase inhibitory action against AChE and BChE in dose-dependent manner using various concentrations (62.5–1000 μg/mL). The IC_50_ values were calculated as 12.13 **±** 0.43 μM against AChE, while 21.41 **±** 0.23 μM against BChE as compared to the standard drugs allanzanthane A and galanthamine (Table 4). Mode of inhibition was determined by Lineweaver-Burk, Dixon plots and replots along with mechanism-based kinetic study which suggested that isotalatizidine hydrate is competitive inhibitor of acetylcholinstrase and butyrylcholinstrase, as there observed an increase in *V*_max_ while decreasing the affinity (*K*_m_ values) of acetylcholinstrase and butyrylcholinstrase towards the acetylthiocholin and butyrylthiocholin, respectively. This means that compound **1** and acetylthiocholin and butyrylthiocholin bind randomly and independently at the active sites of acetylcholinstrase and butyrylcholinstrase. The graphical data on the basis of steady state inhibition for compound **1** against acetylcholinstrase and butyrylcholinstrase has been presented in Figure 3. Based on the aforementioned facts, hence it is concluded that, isotalatizidine hydrate showed significant inhibitory activity against both the enzymes.

**Table 4.** AChE and BChE inhibitory activities of compound **1** (isotalatizidine hydrate).

		AChE ± SEM^a^	BChE ± SEM^a^	
S.No	Compound/Standard	μM	μM	Type of inhibition
1	Isotalatizidine hydrate	12.13**±** 0.43	21.41**±** 0.23	Competitive
2	Allanzanthane A	8.23**±** 0.01	18 **±** 0.06	—
3	Galanthamine^b^	10.12 ± 0.06	20.62 ± 0.08	—

^a^Standard error of mean of five assays.

^b^Positive control used in the assays.

##### Molecular docking studies

The crystal structure of isotalatizidine hydrate was docked into the binding pocket of the enzymes AChE and BChE separately in order to find the binding interactions of the compounds with the protein. For docking studies, the default parameters of the MOE used were i-e Placement: Triangle Matcher, Rescoring 1: London dG, Refinement: Forcefield, Rescoring 2: London dG. For each ligand, 10 conformations were allowed to be formed and the top ranked conformations on the basis of docking score were selected for further analysis. Docking score is the binding free energy calculated by the GBVI/WSA scoring function which is the score of the last stage showing the overall fitness of compound in the pocket. For all scoring functions, lower scores indicate more favourable poses. The unit for all scoring functions is kcal/mol (Alam et al. 2016). The compound was recognized to fit in the binding pocket of the AChE with the docking score −11.6236 and revealed efficient interactions with the active/functional site residues of the receptor protein Tyr 121 and Tyr 334. Almost similar binding pattern was observed in case of BChE in which Trp 82 interacts with hydroxyl group and Thr 120 with methoxy group showing a docking score −13.4501.

## Discussion

**Figure 4.**
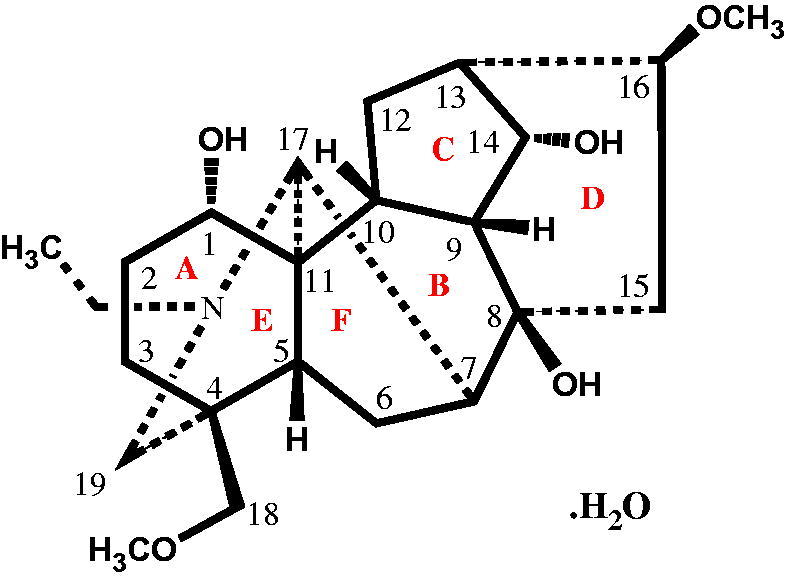
Structure of compound **1**, orientation of different rings and groups are shown.

Isotalatizidine hydrate, a colourless crystalline natural product, consists of six rings (A–F) of different size. Six-membered rings A (C1/C2/C3/C4/C5/C6) and ring E (N1/C9/C4/C5/C6/C13) assumed chair conformation while the six membered ring D (C21/C22/C715/C16/C20/C19) adopted boat conformation. The five-membered rings C (C16/C17/C18/C19/C20) and F (C5/C6/C10/C13/C14) indicated an envelope conformation, whereas ring D (C8/C9/C14/C13/C16/C15) assumed boat conformations. The absolute configuration of compound **1** cannot be confirmed by Mo-Kα diffraction data, but it can be assumed to be the same as reported for C_19_ and C_20_-diterpenoid alkaloids isolated from the natural source (Lei et al. 2011; Tashkhodjaev & Sultankhodjaev 2007), however, compound **1** has two α-oriented hydroxyl substituent at C-1 and C-14 while the two methoxy groups at C-8 and C-16 are β-oriented (Figure 4). On the basis of crystal data, the theoretical calculation of HOMO-LUMO energies and energy gap difference between HOMO and LUMO were also studied. The energy gap between frontier orbitals HOMO-LUMO is 0.220 which shows the stability of LUMO due to the electron accepting properties whereas the value of HOMO is commonly related to the electron donating capacity of inhibitor molecule. HOMO with higher values is a signal of the higher ease of donating electrons to the unoccupied orbital of the receptor. The low band gap and other parameters of the compound are responsible for its high reactivity and low stability (Renuga & Muthu 2014).

Although AChE has major significance over BChE but both the enzymes indirectly sustain one another for their cholinomimetic profile. Based on previous concepts, it is proposed that normal decrease in function of AChE in brain is substituted by normal increase in function of BChE, which may act as an alternative mechanism for ACh hydrolysis. During advanced AD, the regulation of ACh depends on BChE. It was observed in a clinical investigation that patients taking dual cholinesterase inhibitors as medication have shown small cortical atrophic variations as compared to those people who are using AChE as selective inhibitors. This useful indication further encourages the basic role of dual inhibitors (Duysen et al. 2007). It is believed that the natural products are safe as compared to synthetic compounds (Raskin et al. 2002). Due to great interest towards the natural products, the scientists are trying to investigate more and more plants for the presence of effective compounds, which may cure a specific disease. It is obvious of our current investigations that isotalatizidine hydrate possesses comparable anticholinesterase potential as compared to the standard drugs allanzanthane A and galanthamine.

**Figure 5.**
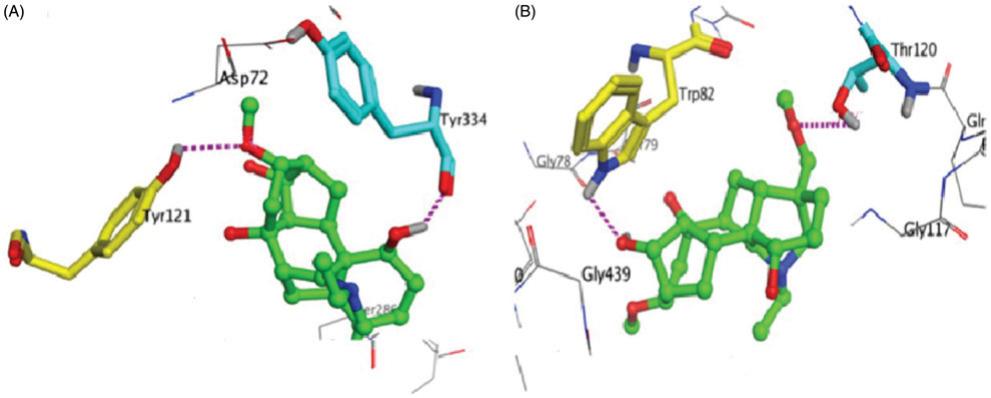
3D binding mode of compound **1** as competitive inhibitor of AChE and BChE.

According to molecular docking, isotalatizidine hydrate was observed that it entered tremendously into the binding pocket of AChE and BChE, showing importance of its molecular shape. Isotalatizidine hydrate displayed notable interactions with essential subsites inside the functional site residues of the receptor protein Tyr 121 and Tyr 334 (Figure 5(A)). Remarkably, hydroxyl groups of isotalatizidine hydrate was observed in contact with Tyr334 through hydrogen bonding. This obviously shows the critical significance of OH group present in isotalatizidine hydrate that allowed the compound to make significant bonding interaction with active site inside the catalytic pocket. Similarly, bonding interaction with Tyr 121 is shown by the methoxy groups of the molecule. Almost similar binding pattern was observed in case of BChE in which Trp 82 interacts with hydroxyl group and Thr 120 with methoxy group, respectively (Figure 5(B)). Finally, the combined effect of hydrogen-bonding due to hydroxyl group of the molecule and methoxy interaction with active site may be attributed to the significant cholinesterase inhibitory activity of isotalatizidine hydrate.

This study summarizes the isolation of potent cholinesterase inhibitors from natural source either in crude or pure form that can be a leading drug for the treatment of Alzheimer’s disease (Zhang 2004). These findings will exponentially increase the interest of this type of compounds found in *Delphinium denudatum* and may prompt chemists towards total synthesis of these compounds for possible commercialization as medicine against Alzheimer’s disease.

## Supporting information

The CIF of the compound has been deposited at the Cambridge Crystallographic Data Center as supplementary publication No. CCDC 1444946. These data can be retrieved free of charge from the Cambridge Crystallographic Data Centre, www.ccdc.cam.ac.uk/getstructures
